# Complete Recovery From Human Herpesvirus 6 (HHV-6) Encephalomyelitis in an Immunocompetent Adult: A Rare Cause of Paraparesis and Ataxia

**DOI:** 10.7759/cureus.90211

**Published:** 2025-08-16

**Authors:** Daya Mani Jacob, Divyashri R Nagarajan, Mohsin Bava, Jawad Fazal, Niyas Khalid Ottu Para

**Affiliations:** 1 Internal Medicine, Burjeel Medical City, Abu Dhabi, ARE; 2 Neurology, Burjeel Medical City, Abu Dhabi, ARE

**Keywords:** acute transverse myelitis (atm), encephalomyelitis, ganciclovir treatment, hhv 6, human herpesvirus type 6 (hhv-6)

## Abstract

Human herpesvirus 6 (HHV-6) is a commonly acquired virus in early childhood, typically resulting in a benign and self-limited illness. However, its neuroinvasive potential in adults, particularly in immunocompetent individuals, remains underrecognized and diagnostically challenging. Clinical features such as nonspecific fever, respiratory symptoms, and evolving neurological deficits can mimic autoimmune demyelination, post-infectious encephalitis, or other viral myelitides, potentially delaying recognition and treatment. We report the case of a previously healthy 32-year-old male who presented with fever, lower respiratory symptoms, and progressive neurological deficits, including lower limb weakness, gait instability, and sensory abnormalities suggestive of spinal cord involvement. Initial imaging was inconclusive, but cerebrospinal fluid analysis revealed lymphocytic pleocytosis, elevated protein, and was positive for HHV-6 by Polymerase Chain Reaction (PCR). The patient was treated with intravenous ganciclovir and immunoglobulin, leading to clinical improvement and viral clearance. This case highlights the importance of considering HHV-6 in the differential diagnosis of acute encephalomyelitis, even in immunocompetent adults, and demonstrates that early antiviral and immunomodulatory therapy can lead to favorable neurological outcomes.

## Introduction

Human herpesvirus 6 (HHV-6)-associated encephalomyelitis in immunocompetent adults is rare, with limited cases reported, and the underlying mechanisms may involve immune dysregulation or direct neuroinvasion [1]. HHV-6, a ubiquitous neurotropic virus, is increasingly recognized for its potential to cause central nervous system (CNS) complications beyond the immunocompromised population.

Clinically, encephalomyelitis often presents with a combination of encephalitic symptoms, such as altered mental status, confusion, seizures, or focal neurological deficits, and myelitis-related features, including limb weakness, sensory loss, or bladder or bowel dysfunction [2].

Although primary infection typically occurs in early childhood, causing roseola infantum, it remains latent after primary infection, and a reactivation or primary infection in adults can result in severe neurological manifestations. Latent HHV-6 is detectable in up to 90-95% of adults, yet CNS reactivation in immunocompetent individuals remains exceedingly rare. While HHV-6 is well-documented in transplant recipients and immunosuppressed individuals, its role in encephalitis and myelitis in immunocompetent hosts remains underrecognized and diagnostically challenging [3,4]. The diagnosis in these cases is often delayed or missed due to nonspecific symptoms, subtle imaging findings, and the rarity of reported cases. Encephalomyelitis, defined as inflammation of both the brain and spinal cord, presents diagnostic complexity. A systematic review emphasized the diagnostic complexity in their review of HHV-6 encephalitis in immunocompetent adults, highlighting the need for greater clinical awareness and timely use of CSF PCR testing [5].

Here, we present a case of HHV-6-associated encephalomyelitis in a previously healthy adult male, which illustrates both the diagnostic challenges and potential for recovery with prompt antiviral and immunomodulatory treatment.

## Case presentation

A 32-year-old previously healthy male presented to the emergency department with a one-week history of fever reaching 38.9°C and a productive cough. He had been started on antibiotics at an offshore clinic prior to presentation. Upon reassessment in the hospital, he reported sudden-onset dizziness along with progressive lower limb weakness and difficulty with standing or walking. He described a sensation of swaying from side to side. On examination, he was alert but appeared lethargic with a Glasgow Coma Scale score of 15. Cranial nerve examination was normal, with no facial, bulbar, or ocular movement deficits. Motor strength in the upper limbs was normal (grade 5), although there was mild dysmetria on finger-to-nose testing. The heel-to-shin test was abnormal. The patient had bilateral lower limb weakness graded at 4 and bilateral ankle clonus. Plantar reflex was extensor on the right and equivocal on the left, suggesting upper motor neuron involvement with mild asymmetry in corticospinal tract signs. A sensory level was noted bilaterally around T8 to T10. Coordination was impaired, with a wide-based gait and mild truncal ataxia.

**Figure 1:**
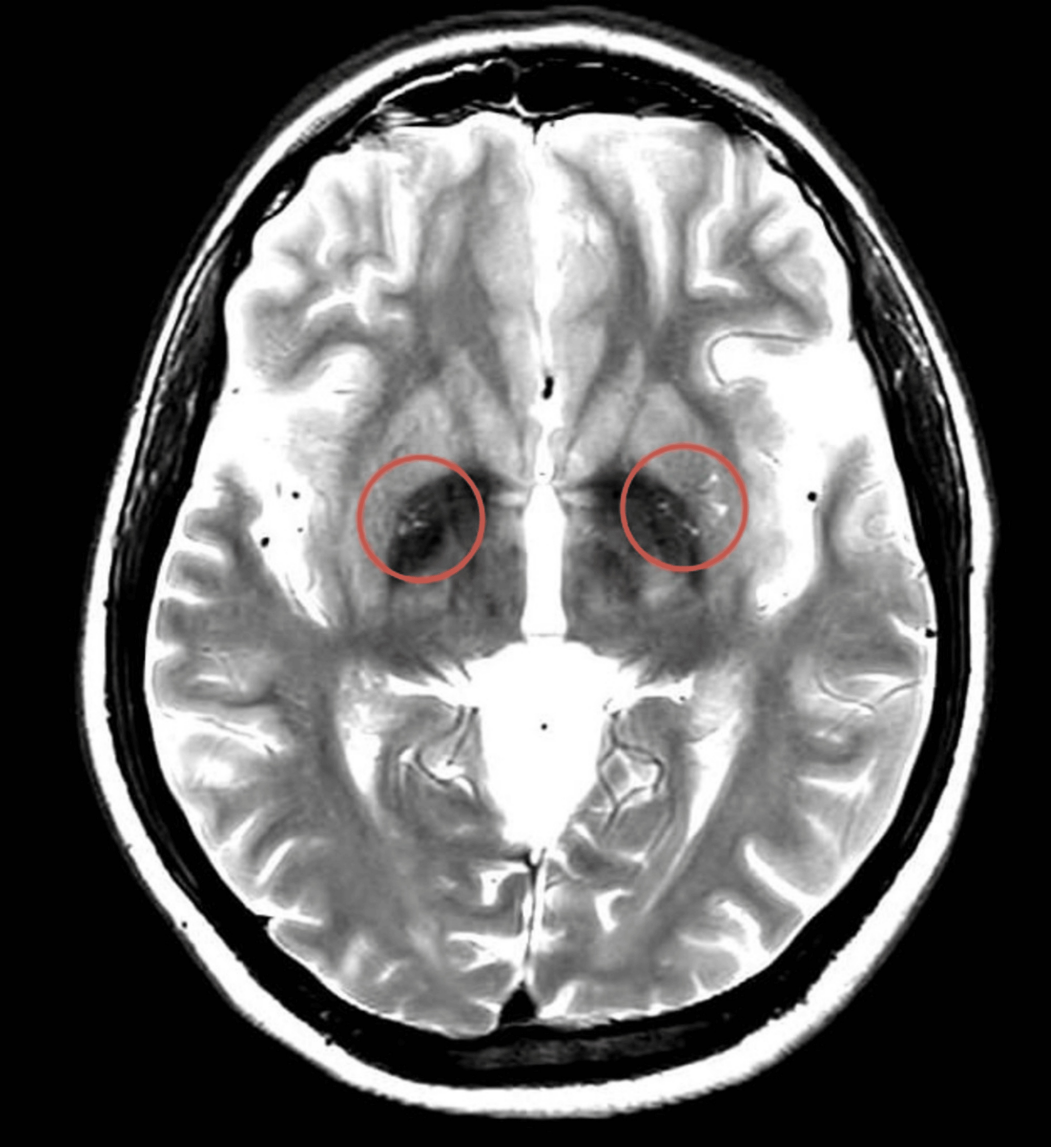
MRI brain showed a few nonspecific white matter hyperintensities

**Figure 2:**
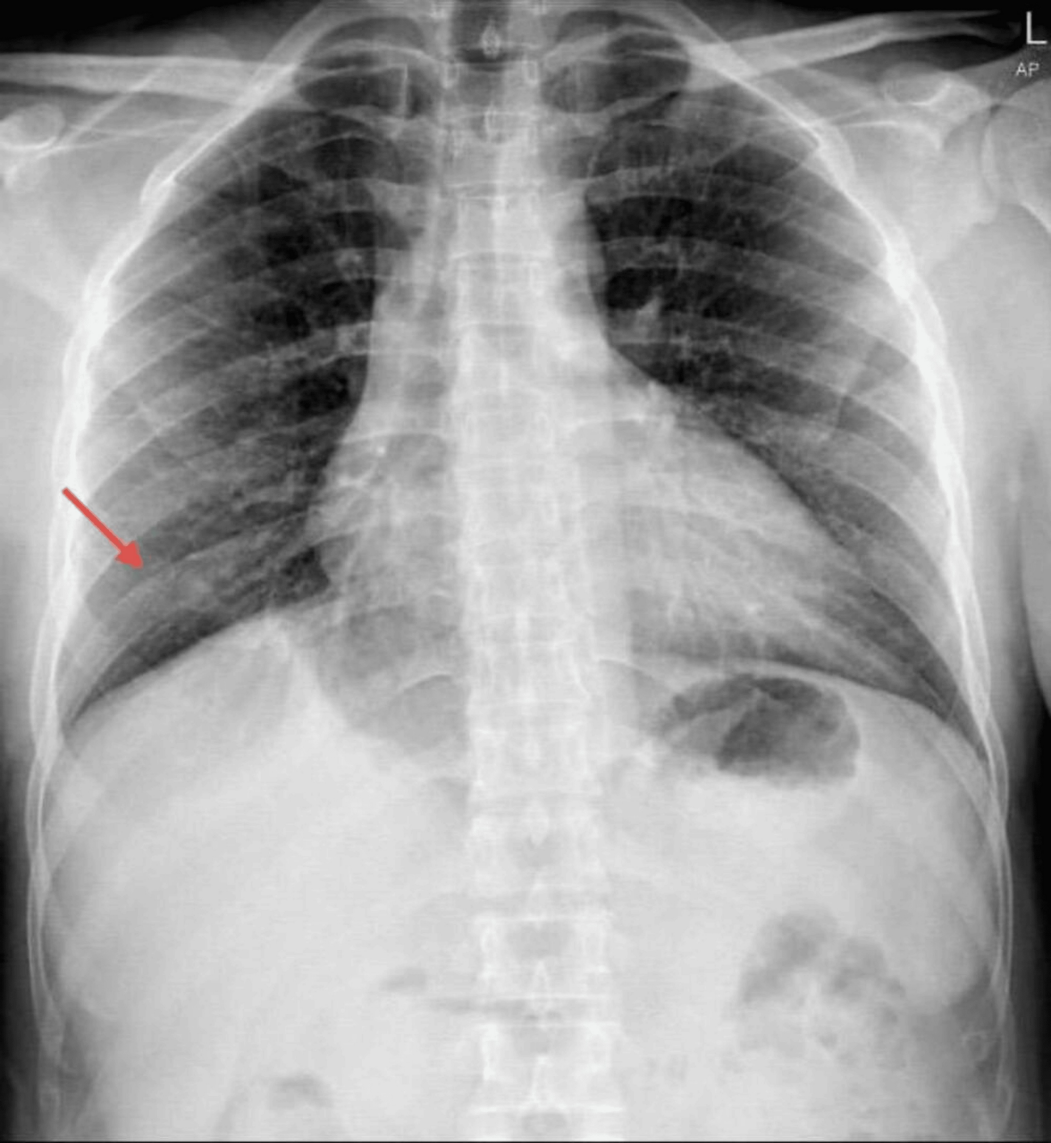
Chest X-ray - Patchy consolidation in the right lower lobe

**Figure 3:**
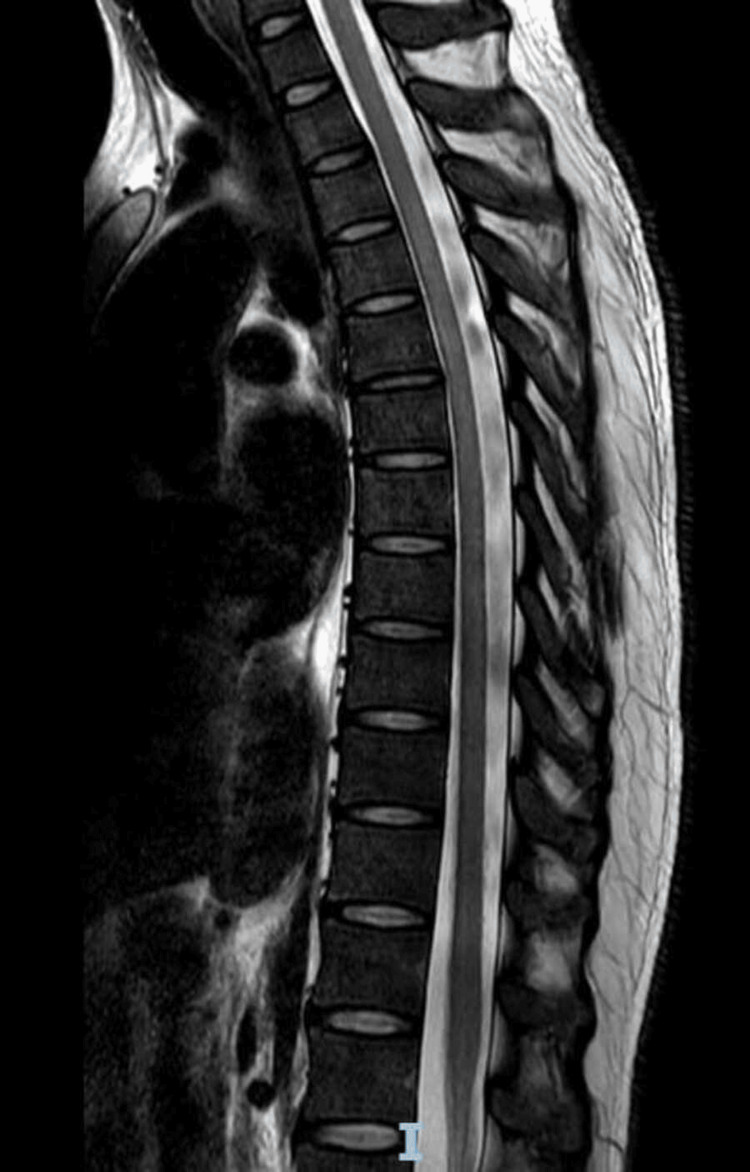
MRI thoracic spine (Sagittal view) - Normal marrow signal and no other significant abnormality

**Figure 4:**
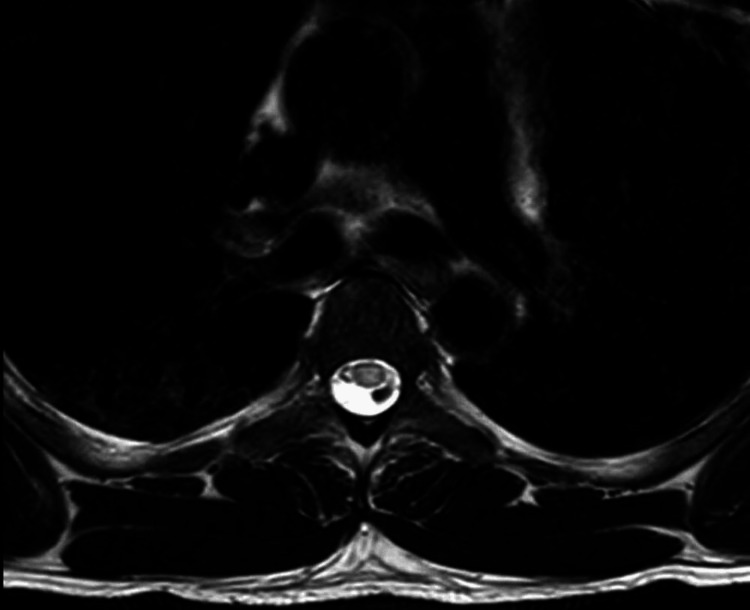
MRI Thoracic spine - Axial view showing no abnormal enhancement

Initial investigations included an MRI of the brain and spine. MRI brain (Figure 1) was largely unremarkable except for a few nonspecific white matter hyperintensities. CT angiography of the brain showed no vascular abnormalities. A chest X-ray (Figure 2) revealed patchy consolidation in the right lower lobe. MRI thoracic spine - sagittal view (Figure 3) and axial view (Figure 4) - was normal. 

An extensive workup was performed to rule out alternative etiologies. Serologic testing was negative for HIV-1 and 2, hepatitis B and C, syphilis (Rapid Plasma Reagin (RPR) and Venereal Disease Research Laboratory (VDRL), *Mycoplasma pneumoniae*, and HTLV-1/2. Autoimmune and inflammatory markers, including antinuclear antibodies (ANA), extractable nuclear antigens, anti-dsDNA, antineutrophil cytoplasmic antibodies (ANCA), and anticardiolipin antibodies (IgM and IgG), were all within normal limits or negative. Epstein-Barr virus (EBV), cytomegalovirus (CMV), and enterovirus testing all returned negative results. Vitamin B12 levels were normal. Cerebrospinal fluid analysis (Table 1) showed no oligoclonal bands. Neuromyelitis optica spectrum disorders were excluded with negative anti-aquaporin-4 (NMO-IgG) and anti-myelin oligodendrocyte glycoprotein (MOG) antibodies. Serum angiotensin-converting enzyme (ACE) levels were also within normal limits. 

**Table 1:** CSF Analysis (Day 1)

Parameter	Value	Reference Range
WBC	345/μL (90% lymphocytes)	0–5
Protein	1.37 g/L	0.15–0.45
Glucose	3.05 mmol/L	2.2–3.9
HHV-6 PCR	Positive	Negative

Based on the neurological presentation with upper limb dysmetria, truncal and gait ataxia (clinically suggesting brain parenchymal involvement), sensory level at thoracic spine with brisk lower limb reflexes (suggesting spinal cord involvement), CSF findings, positive HHV-6 PCR with all other investigations being negative, a diagnosis of HHV-6-associated encephalomyelitis was made.

The patient was managed in consultation with the infectious diseases and neurology teams. Treatment was initiated with intravenous ganciclovir at a dose of 5 mg/kg every 12 hours for 7 days, along with intravenous immunoglobulin (IVIG) at 0.4 g/kg/day for five days. A 7-day course was selected based on rapid recovery, lack of immunosuppression, and to limit ganciclovir toxicity, recognizing that reported durations vary widely.

Repeat CSF analysis showed viral clearance and resolving inflammation (Table 2). CSF parameters improved in parallel with the patient’s neurological recovery, with lymphocytic pleocytosis and protein levels declining, and HHV-6 PCR converting to negative by day 8. Over the course of treatment, CSF analysis showed marked improvement, with WBC count decreasing from 345/μL to 73/μL, protein levels falling from 1.37 g/L to 0.53 g/L, stable glucose levels, and clearance of HHV-6 DNA by PCR.

**Table 2:** Repeat CSF Analysis (Day 8)

Parameter	Value	Reference Range
WBC	73/μL (lymphocytic)	0–5
Protein	0.53 g/L	0.15–0.45
Glucose	3.38 mmol/L	2.2–3.9
HHV-6 PCR	Negative	Negative

Daily complete blood counts were monitored for ganciclovir-induced cytopenia, but no hematological abnormalities or any other adverse effects were observed. Bowel and bladder functions remained intact throughout the admission. Early physical therapy was started, including bed-based strength exercises and gradual ambulation training. By day 7, the patient’s lower limb strength had improved to 5/5, deep tendon reflexes had normalized to ++, and a normal plantar response. However, gait remained mildly unsteady with a wide-based stance and difficulty performing tandem walking.

The patient was discharged after 10 days, ambulant with minimal unsteadiness and advised to continue home physiotherapy. He was instructed to repeat CBC and renal function testing within three days and to follow up in the neurology outpatient clinic within a week. At the four-week follow-up, he remained clinically stable with no new symptoms or relapse reported. He is a skilled electrician, and within 3 months returned to regular work.

## Discussion

HHV-6 is a widespread virus that typically establishes latency after primary infection in early childhood. A recent systematic review by Webb et al. discussed the diagnostic challenges of HHV-6 encephalitis in immunocompetent adults, emphasizing the wide clinical variability, often normal neuroimaging findings, and the reliance on CSF PCR for definitive diagnosis [5]. While it is most often associated with febrile illnesses in infants or reactivation in immunocompromised patients, HHV-6 can also lead to significant CNS disease in immunocompetent adults, a phenomenon that remains underrecognized [6]. Although encephalitis is the most reported manifestation, HHV-6-associated myelitis or encephalomyelitis is rarely described in patients without immunosuppressive conditions.

In transplant recipients, particularly those undergoing allogeneic hematopoietic stem cell transplantation (HSCT), HHV-6 reactivation occurs in 30-70% of patients and can lead to devastating complications such as limbic encephalitis, bone marrow suppression, or graft-versus-host disease [7]. Reactivation typically occurs within 2-4 weeks post-transplant, often presenting with subacute neurocognitive or behavioral symptoms [8,9]. Our patient, however, had no history of transplant or known immunosuppression, suggesting either primary infection or rare reactivation in an otherwise healthy host. While HHV-6 reactivation is uncommon in immunocompetent individuals, it has been reported in association with transient factors such as recent viral illnesses, significant physiological stress, or subclinical immune dysregulation, which may lower host defenses and permit viral reactivation [10].

Another diagnostic difficulty is the possibility of chromosomally integrated HHV-6 (ciHHV-6). ciHHV-6 is the integration of HHV-6 DNA in the telomere of the host cell chromosome, and this can lead to incidental detection of HHV-6 by PCR in the CSF and/or blood of patients [11]. ciHHV-6 will generally result in a persistent positive result in CSF/Blood and generally would not be associated with a significantly abnormal CSF cell count and high CSF protein, indicating active inflammation, as was the case in our patient, unless there is active HHV-6 replication, reactivation, or there is a co-existing unidentified alternative viral etiology [12].

Our patient presented with progressive neurological symptoms following a nonspecific febrile illness and had initially inconclusive neuroimaging. Subtle abnormalities on brain MRI, in conjunction with significant CSF lymphocytic pleocytosis (345 WBC with 90% lymphocytes), elevated protein, and positive HHV-6 PCR in the absence of any other identified organism/etiology, supported the diagnosis of HHV-6-associated encephalomyelitis. The absence of oligoclonal bands made a demyelinating disorder such as multiple sclerosis less likely, while the marked lymphocytic pleocytosis supported a viral or post-infectious inflammatory etiology.

We tested HHV-6 quantitative PCR in the whole blood sample for our patient, which was negative. This also makes ciHHV-6 less likely. Importantly, whole blood HHV-6 viral loads exceeding approximately 5.5 log₁₀ copies/mL are strongly suggestive of chromosomally integrated HHV-6 (ciHHV-6), and such cases warrant confirmatory testing such as hair follicle PCR to definitively rule out ciHHV-6 [13]. Moreover, after active treatment, our patient’s CSF became negative for HHV-6 with significant improvement in CSF inflammatory response (reduced cell count and protein).

Encephalomyelitis due to HHV-6 in immunocompetent adults is increasingly being reported [6]. Many cases, along with ours, suggest that early antiviral and immunomodulatory therapy can be effective even in immunocompetent patients [14].

Among the available antiviral options, ganciclovir is frequently used as first-line therapy for HHV-6 CNS infection due to its proven in‑vitro activity against viral DNA polymerase, while foscarnet serves as an alternative, particularly in cases of intolerance or resistance to ganciclovir [9]. Although randomized controlled trials are lacking, the 2017 European Conference on Infections in Leukaemia (ECIL) guidelines highlight observational evidence in HSCT recipients showing that prompt initiation of either agent may decrease viral load and improve neurological outcomes [15]. IVIG or corticosteroids are used in HHV-6 encephalomyelitis to mitigate immune-mediated inflammation, similar to that seen in acute disseminated encephalomyelitis (ADEM). Corticosteroids suppress pro-inflammatory cytokines and lymphocyte activation, while IVIG modulates immune responses through Fc receptor blockade, neutralization of autoantibodies, and inhibition of complement activation, particularly in steroid-refractory cases [1]. In our case, the combination of ganciclovir and IVIG led to clinical and virological improvement within one week, with no hematologic toxicity and complete resolution of CSF abnormalities on repeat testing.

This case is notable for the complete neurological recovery following early initiation of combined antiviral (ganciclovir) and immunomodulatory (IVIG) therapy, commenced within the first week of neurological symptom onset. The patient demonstrated thoracic-level sensory findings (T8-T10) and brisk reflexes suggestive of spinal cord involvement despite a normal spinal MRI, a presentation that is rarely documented in HHV-6 encephalomyelitis. Early dual therapy, preserved sphincter function, and rapid functional improvement, with return to baseline mobility within weeks, emphasize the potential for favorable outcomes when prompt diagnosis and treatment are achieved in immunocompetent individuals.

Additionally, several important clinical lessons emerge from this report. First, HHV-6 should be considered in the differential diagnosis of encephalomyelitis or myelitis even in patients without immunosuppression. Second, MRI may be normal or subtle in early disease, necessitating expert review and the use of CSF PCR for diagnosis. Third, prompt initiation of antiviral therapy, with or without immunomodulation, can lead to excellent outcomes if started early. Finally, although most treatment guidelines are based on transplant populations, the same principles may be applicable in select immunocompetent individuals presenting with CNS involvement. Given the limited number of reported cases and absence of standardized treatment protocols, continued reporting and prospective studies are needed to guide clinical practice in immunocompetent patients.

## Conclusions

This case adds to the growing body of literature recognizing HHV-6 as a potential cause of myelitis in immunocompetent adults. Its distinctiveness lies in the absence of prior immunosuppression, the presence of spinal cord symptoms despite normal MRI findings, and the rapid, complete neurological recovery following early combined antiviral (ganciclovir) and immunoglobulin therapy. In patients presenting with acute spinal cord dysfunction and recent infection, clinicians should consider HHV-6 as a differential. Timely CSF analysis, early antiviral therapy, and supportive rehabilitation can yield excellent recovery. The rapid functional improvement observed in this case suggests the potential for favorable outcomes when diagnosis and treatment are initiated promptly, even in the absence of radiological abnormalities. This case, therefore, adds to the limited literature on HHV-6 encephalomyelitis in immunocompetent adults and emphasizes the need for early CSF analysis and antiviral therapy for favorable outcomes.
